# Molecular and immunological characterization of allergens from the entomopathogenic fungus *Beauveria bassiana*

**DOI:** 10.1186/1476-7961-4-12

**Published:** 2006-09-22

**Authors:** Greg S Westwood, Shih-Wen Huang, Nemat O Keyhani

**Affiliations:** 1 Department of Microbiology and Cell Science, University of Florida, Gainesville, FL 32611, USA; 2 Department of Pediatrics, University of Florida, College of Medicine, 32610, USA

## Abstract

**Background:**

Entomopathogenic fungi such as *Beauveria bassiana *are considered promising biological control agents for a variety of arthropod pests. *Beauveria *species, however, have the potential to elicit allergenic reactions in humans, although no specific allergens have been characterized to date.

**Methods:**

Four putative allergens were identified within *B. bassiana *expressed sequence tag (EST) datasets. IgE-reactivity studies were performed using sera from patients displaying mold allergies against recombinant *B. bassiana *proteins expressed in *E. coli*.

**Results:**

Full length cDNA and genomic nucleotide sequences of four potential *B. bassiana *allergens were isolated. BLASTX search results led to their putative designation as follows; Bb-Eno1, with similarity to fungal enolases; Bb-f2, similar to the *Aspergillus fumigatus *major allergen, Asp f2 and to a fibrinogen binding mannoprotein; Bb-Ald, similar to aldehyde dehydrogenases; and Bb-Hex, similar to N-acetyl-hexosaminadases. All four genes were cloned into *E. coli *expression systems and recombinant proteins were produced. Immunoblots of *E. coli *extracts probed with pooled as well as individual human sera from patients displaying mould allergies demonstrated IgE reactivity versus recombinant Bb-Eno1 and Bb-Ald.

**Conclusion:**

Four putative *Beauveria bassiana *allergens were identified. Recombinant proteins corresponding to two of the four, Bb-Eno1 and Bb-Ald were bound by sera IgEs derived from patients with fungal allergies. These data confirm the potential allergenicity of *B. bassiana *by identification of specific human IgE reactive epitopes.

## Background

Allergic diseases represent a growing human health problem, affecting up to 25% of individuals living in industrialized nations [1]. Both in- and outdoor populations of filamentous fungi are a major cause of human allergies and asthma, and can in some cases, lead to severe allergic disease [2]. Overall, some 30% of asthma cases can be attributed to exposure and sensitization to filamentous fungal allergens [3-5].

*Beauveria bassiana *is an entomopathogenic fungi currently under intensive study as a biological control agent against a wide range of agricultural, nuisance, and disease carrying insect pests [6-10]. *B. bassiana *is considered non-pathogenic to vertebrates, has not been deemed a potential health or environmental hazard [11], and has received EPA approval for commercial use. Volumetric assays of allergens performed in the Netherlands in the 1980's, revealed that although the environmental concentration of *Beauveria *spores was very low, the allergic response was quite high [12,13]. Using skin prick assays on patients with mold allergies, *B. bassiana *was shown to elicit one of the strongest reactions relative to the other fungal species tested. More recently, it has been confirmed that crude extracts of *B. bassiana *can elicit allergic reactions in humans [14]. Sera IgEs derived from patients displaying allergies to molds as well as from people with no known allergies reacted with several proteins present in *B. bassiana *crude extracts. Many of these proteins were cross reactive with epitopes present in a number of major allergenic fungi, however the identities of any specific *B. bassiana *allergen has yet to be reported. In order to gain more information concerning *B. bassiana *and its potential allergenicity it is important to isolate the genes coding for IgE-binding allergens and characterize their protein products. Recombinant purified allergens, as compared to crude fungal extracts, can then be used to examine the nature of the IgE binding as well as in the diagnosis of allergy, in that the recombinant proteins are more standardized, can be highly purified, and hence are more suitable for immunodiagnosis [15,16].

A significant number of fungal allergens are proteins of unknown function, although the biochemical activities of a number of allergens have been characterized. These typically fall into several classes including metabolic enzymes, proteases, and enzyme inhibitors [5,17]. A molecule identified as an allergen in one species of fungus is often found to be an allergen when identified in other species, presumably due to similarities in structure and hence IgE-reactive epitopes. Thus, aldehyde dehydrogenase has been identified as an allergen in both *Alternaria alternata *(Alt a10) and *Cladosporium herbarum *(Cla h3) [18]. Amongst other metabolic enzymes, enolases (2-phosho-D-glycerate hydrolase) from a wide range of organisms, are common allergens with shared epitopes [19-21]. This phenomenon of cross-reactivity of an IgE produced in response to an antigen from one organism to another can lead to wide spectrum allergic reactions derived from the original sensitization [22-24].

Here we report the identification of four *B. bassiana *proteins as potential allergens. Full length cDNA and genomic nucleotide sequences of the four genes were determined. Similarity search results of the translated open reading frames of the proteins coded by the genes have led to their putative designation as follows; Bb-Eno1, an enolase; Bb-Ald, aldehyde dehydrogenase; Bb-f2, similar to Asp f2 and a fibrinogen binding mannoprotein; and Bb-Hex, an N-acetylhexosaminidase. The cDNA sequences of the proteins were used to design primers for subcloning of the genes into *E. coli *expression vectors. All four proteins were expressed as recombinant proteins in *E. coli*. Two of these proteins, Bb-Eno1 and Bb-Ald reacted with human IgEs derived from patients displaying mold allergies.

## Methods

### Strains and cultures

*Beauveria bassiana *(ATCC 90517) was maintained on Potato dextrose (PD) agar at 26°C. *E. coli *stains TOPO Top10 (Invitrogen, CA) and BL21 Rosetta (DE3), harboring the pRARE plasmid (Novagen, Darmstadt, Germany) were used for routine cloning and protein expression, respectively. *E. coli *strains were grown in Luria-Bertani (LB) nutrient broth or agar plates supplemented with the appropriate antibiotics as indicated.

### Bioinformatic identification of putative allergen genes

Construction and sequencing of expressed sequence tagged (EST) cDNA libraries derived from five different developmental stages of *B. bassiana *has recently been reported [25,26]. Additional sequences were obtained by suppressive subtractive hybridization (SSH) using fungal cells grown on insect cuticles and fungal cells grown on glucose as the tester and driver mRNAs respectively using established protocols [27,28]. BLASTX similarity searches using the sequence dataset (~18,000 ESTs) revealed four sequences with high homology to allergen genes.

### Molecular manipulations

Molecular manipulations including plasmid isolation, restriction digestion, agarose-gel electrophoresis, and PCR were performed using standard methods. Template mRNA was extracted from *B. bassiana *grown on minimal medium (per L; 0.4 g KH_2_PO_4_, 1.4 g Na_2_HPO_4_, 0.6 g MgSO_4_-7H_2_O, 1.0 g KCl, 0.25 g NH_4_NO_3_, 0.01 mg FeSO_4_) supplemented with 0.1% N-acetylglucosamine and 10% sterilized insect cuticle (mole cricket, *Scapteriscus abbreviatus*). Cultures were inoculated with 10^5 ^conidia/ml and grown with aeration for 6 d at 25°C. Fungal cells were lysed by grinding in liquid nitrogen and total RNA was extracted using RNAWiz (Ambion). cDNA libraries were constructed using the SMART RACE cDNA Amplification kit (Clontech, CA) according to manufacturer instructions. For construction of *E. coli *expression plasmids, an NdeI restriction site was incorporated into the forward primer and an EcoRI site into the reverse primer. PCR products were cloned directly into TOPO 2.1 using TOPO TA cloning system and transformed into TOPO Top 10 *E. coli *cells (Invitrogen, Carlsbad, CA). The TOPO 2.1 constructs were used for subcloning into the NdeI-EcoRI sites of pET43.1a (Novagen, Darmstadt, Germany) for expression using *E. coli *BL21 host strain harboring the pRARE plasmid.

### Protein expression, Western and immunoblotting

Overnight cultures of *E. coli *BL21 harboring pRARE along with each respective pET43.1a based construct were grown in 3 ml of LB (supplemented with 50 μg/ml ampicillin and 12 μg/ml chloramphenicol) at 37°C with aeration. Fresh media (5–10 ml) was inoculated with aliquots (0.1–0.2 ml) of the overnight culture, and samples were incubated at 37°C with aeration to an OD_600 _= 0.6–0.8. T7 polymerase based expression of the recombinant proteins was initiated by the addition of 1–1.5 mM (final concentration) isopropyl-β-D-thiogalactopyranoside (IPTG), and cultures were returned to the incubator for an additional 2–3 hours. For extract preparation, cells were harvested by centrifugation (10,000 × g, 10 min) and the resultant pellet resuspended in 0.5 volumes TE (40 mM Tris, 1 mM EDTA, 0.01% phenylmethylsulfonyl fluoride (PMSF)). Cells were lysed by sonication (3 × 30 sec) on ice, after which samples were centrifuged (10,000 × g, 10 min) and separated into soluble and pellet (containing potential inclusion bodies) fractions. Samples of the crude soluble and pellet extracts were denatured with 4× LDS loading dye (Invitrogen) and boiled for 1–5 min prior to separation by SDS-Polyacrylamide gel electrophoresis (PAGE) using the Invitrogen NUPage-MOPS buffer system (10–12% Bis-tris polyacrylamide gels) according to the manufacture's recommended protocols. Gels were stained with Coomasie Blue R250 followed by destaining with 10% methanol, 10% acetic acid solution. For Western blots and immunodetection, samples were analyzed by SDS-PAGE as described above, followed by electroblotting to polyvinylidene-fluoride (PVDF) membranes (Invitrogen). After blocking (TBST; 25 mM Tris-HCl buffer saline containing 0.1% Tween-20 and 10% dry fat free milk), membrane were probed with either individual or pooled human sera as the primary antibody solution. Typically, sera were diluted in blocking buffer and incubated with membranes overnight at 4–8°C with gentle agitation. Membranes were washed 3 × using 50 ml TBST for 15 min each. Binding of human IgEs was visualized using a horseradish peroxidase (HRP) conjugated goat anti-human IgE (polyclonal) secondary antibody (BioSource International, CA). Membranes were incubated in secondary antibody (diluted 1:10,000 in blocking buffer) for 1 hr at room temperature, with gentle agitation. After secondary antibody incubation membranes were washed 3 times using 50 ml TBST and bands visualized using the Immuno-Star HRP detection system (Bio-Rad, Hercules, CA). Total protein membrane staining was performed using Ponceau S (Sigma, St. Louis, MO).

### Analysis programs

Nucleotide manipulations and phylogenetic analyses were performed using multiple software programs. Initial sequence alignments were performed with ClustalW [29]. Alignment files (in Nexus format) were transferred to Splitstree for analysis and construction of phylograms, with typical bootstrap parameters set to 1000 [30].

### Genbank submission

The isolated cDNA and genomic sequences of the four *B. bassiana *genes have been submitted to Genbank with the following accession numbers; Bb-Eno1, DQ767719; Bb-f2, DQ767720; Bb-Ald, DQ767722; and Bb-Hex, DQ767722.

## Results

### Molecular characterization of four putative *B. bassiana *allergens

EST (Expressed sequence tag) panning and screening of a suppressive subtractive library (SSH) identified gene fragments of four potential allergens by sequence homology. The *B. bassiana *genes were designated as follows: Bb-Eno1, similar to *Cladosporium herbarum *enolase Cla h 6 [18]; Bb-f2, similar to *Aspergillus fumigatus *major allergen Asp f2 [31]; Bb-Ald, similar to *C. herbarum *allergen Cla h 3, an aldehyde dehydrogenase [18]; and Bb-Hex, with similarity to numerous fungal N-acetylhexosaminidases, including the *Penicillium chrysogenum *Pen ch 20 allergen [32].

Since the nucleotide fragments (200–300 bp) represented only a portion of the entire gene sequence coding for each protein, full length sequences were obtained by 5' and 3' RACE PCR as needed. These results were used to assemble the full length cDNA nucleotide sequences of the four genes. Separate sets of primers were then designed for amplification of the genomics DNA sequences of the genes and for cloning into the *E. coli *pET43a-based protein expression system as described in the Methods section. The lengths of the cloned cDNA and genomic sequences, the number of introns, along with an analysis of the predicted ORFs, detailing the number of amino acids, molecular mass, and pIs of the deduced *B. bassiana *proteins are given in Table 1. Top BLASTX search results for each protein are also presented (Table 2).

**Table 1:** Characteristics of the cloned *B. bassiana *genes and their predicted protein products

Protein ID	putative function	genomic clone (bp)	# of introns	cDNA clone (bp)	# of amino acids	Molecular mass (KDa)	pI (protein)
Bb-Eno1	Enolase	1548	4	1317	438	47.4	5.07
Bb-f2	Unknown	845	1	786	261	28.6	7.64
Bb-Ald	aldehyde- dehyrogenase	1659	2	1494	497	53.9	5.99
Bb-Hex	hexosaminidase	1959	0	1959	652	72	5.56

**Table 2:** BLASTX search results using full-length *B. bassiana *sequences

Query	Search Results				
	Organism	Function	Allergen I.D.	Accession number	E-value
Bb-Eno1				DQ767719	
	*Alternaria alternata*	enolase	Alt a 6	U82437	<10^-100^
	*Cladosporium herbarum*	enolase	Cla h 6	X78226	<10^-100^
	*Aspergillus fumigatus*	enolase	Asp f 22w	AF284645	<10^-100^
	*Neurospora crassa*	enolase	-^1^	XM323150	<10^-100^
	*Penicillium citrinum*	enolase	Pen c 22w	AF254643	<10^-100^
Bb-f2				DQ767720	
	*Aspergillus fumigatus*	major allergen	Asp f 2	AAC69357	10^-64^
	*Aspergillus nidulans*	antigen 1	-	XP659435	10^-55^
	*Candida albicans*	pH regulated antigen	-	AAC00525	10^-52^
	*Candida albicans*	fibrinogen binding mannoprotein	-	AAC49898	10^-52^
Bb-Ald				DQ767721	
	*Alternaria alternata*	aldehyde dehydrogenase	Alt a 10	X78227	<10^-100^
	*Cladosporium herbarum*	aldehyde dehydrogenase	Cla h 3	X78228	<10^-100^
	*Cladosporium fulvum*	aldehyde dehydrogenase	-	AF275347	<10^-100^
	*Neurospora crassa*	aldehyde dehydrogenase	-	XM951769	<10^-100^
	*Aspergillus nidulans*	aldehyde dehydrogenase	-	XM653066	<10^-100^
Bb-Hex				DQ767722	
	*Metarhizium anisopliae*	N-acetylhexosaminidase	-	DQ000319	<10^-100^
	*Aspergillus fumigatus*	N-acetylhexosaminidase	-	XM742214	<10^-100^
	*Aspergillus oryzae*	N-acetylhexosaminidase	-	AB085840	<10^-100^
	*Penicillium chrysogenum*	N-acetylhexosaminidase	Pen ch 20	AAB34785	10^-47^

^1 ^Dash indicates that it is unknown whether the protein is an allergen.

The genomic sequence of Bb-Eno1 consisted of 1548 bp from the start site to the stop codon and contained four introns. The lengths of the introns were between 52–69 bp and were located in the first half of the gene. The cDNA sequence of the open reading frame of Bb-Eno1 consisted of 1317 bp, constituting a protein of 438 amino acids with a calculated molecular mass ~47 kDa. BLASTX similarity searches of the complete Bb-Eno1 amino acid sequence against the NCBI protein database confirmed the initial observation, resulting in high similarity to enolases derived from numerous fungal species, including *A. fumigatus*, *Penicillium citrinum*, *Alternaria alternata*, and *C. herbarum*.

The genomic sequence of Bb-f2 consisted of 845 bp (start to stop codon) and contained one intron that began at bp 412 and was 59 bp in length. The coding sequence of Bb-F2 consisted of 261 amino acids, with a calculated molecular mass of 28 kDa. BLASTX similarity searches confirmed that Bb-f2 displayed high sequence similarity to the *A. fumigatus *major allergen Asp f 2.

The Bb-Ald genomic clone contained two introns; the first 106 bp in length, 62 bp from the ATG start codon, and the second, 59 bp in length, starting 568 bp from the start codon. The total size of the genomic clone was 1659 bp (start to stop codon), with the cDNA sequence consisting of 1494 bp coding for a proteins comprised of 497 amino acids with a calculated molecular mass of 53 kDa. BLASTX similarity searches using the complete Bb-Ald sequence as the query revealed similarity to aldehyde dehydrogenases, including those from *A. alternata *and *C. herbarum*.

The genomic clone corresponding to Bb-Hex was 1959 bp in length (start to stop codon) and did not contain any introns. The open reading frame coded for a protein consisting of 652 amino acids with a calculated molecular mass of 72 kDa. BLASTX similarity searches confirmed high sequence similarity to fungal N-acetylhexosaminidases.

### Expression of recombinant *B. bassiana *proteins

**Figure 1:**
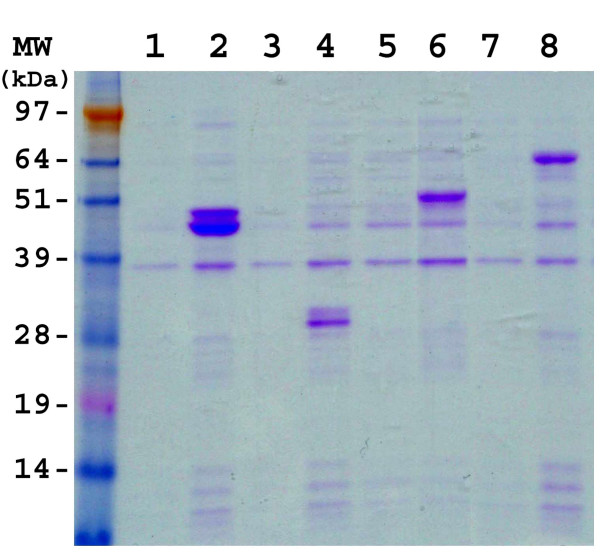
SDS-PAGE analysis of *B. bassiana *recombinant proteins expressed in *E. coli*. SDS-PAGE, Coomasie Blue stained, extracts of *E. coli *strain BL21 harboring pRARE and the indicated expression plasmid constructs; lanes 1) and 2); pET41a:Bb-Eno1, lanes 3) and 4) pET41a:Bb-f2, lanes 5) and 6) pET41a:Bb-Ald, lanes 7) and 8) pET41a:Bb-Hex. Uninduced cell cultures, lanes 1), 3), 5), and 7). IPTG induced cell cultures, lanes 2), 4), 6), and 8).

**Figure 2:**
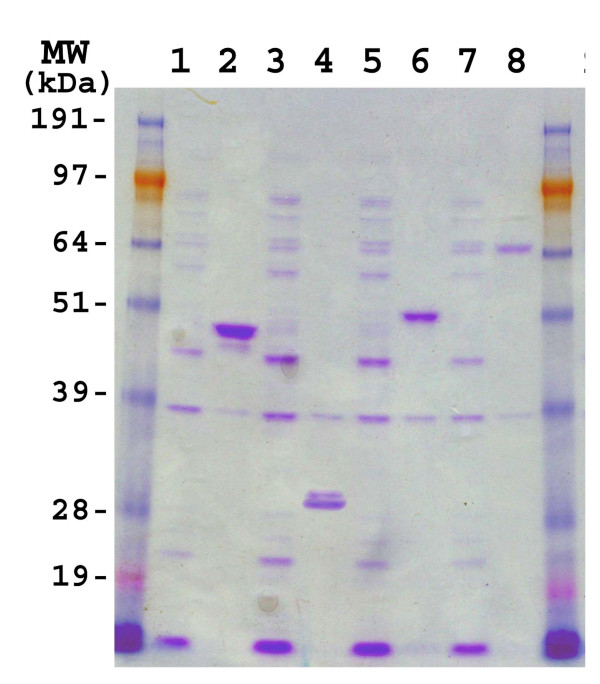
SDS-PAGE analysis of soluble and pellet (inclusion bodies) fractions of the *B. bassiana *proteins expressed in *E. coli*. SDS-PAGE, Coomasie Blue stained extracts of soluble fractions lanes 1), 3), 5) and 7), and pellet fractions, lanes 2), 4), 6), and 8). Expression of Bb-Eno1, lanes 1) and 2), Bb-f2, lanes 3) and 4), Bb-Ald, lanes 5) and 6), and Bb-Hex, lanes 7) and 8).

**Figure 3:**
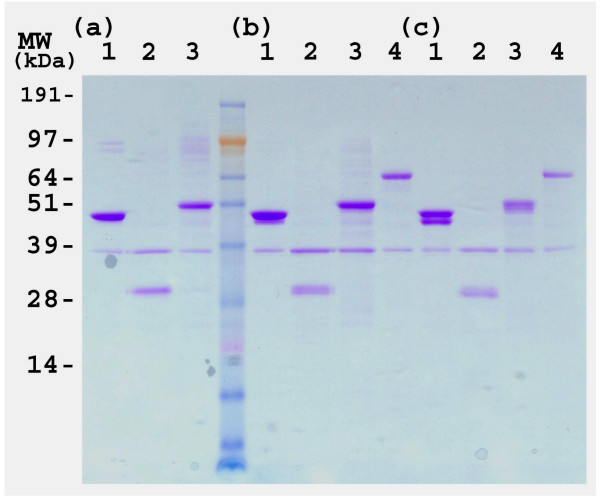
SDS-PAGE analysis of the temperature sensitivity of the recombinant *B. bassiana *proteins. SDS-PAGE, Coomasie Blue stained, *E. coli *crude extracts subjected to; 1 min heat denaturation at 95°C, panel A), 5 min, 95°C, panel B), and 20 min, 95°C, panel C), Lanes correspond to crude extracts containing, lane 1) Bb-Eno1, lane 2) Bb-f2, lane 3) Bb-Ald, and lane 4) Bb-Hex.

The coding sequences of the four *B. bassiana *genes were subcloned into the pET43.1a expression vector as described in the Methods. The integrity of all clones was verified by sequencing of the inserts. The recombinant *B. bassiana *proteins were expressed in *E. coli *strain BL21 harboring the pRARE plasmid that contains the genes for the expression of rare tRNAs (Fig. 1, initial experiments using a BL21 strain lacking the pRARE plasmid resulted in little to no expression). Fractionation of the crude extracts into soluble and insoluble (presumably inclusion bodies) fractions revealed the *B. bassiana *proteins to be largely in the insoluble fraction (Fig. 2). In some instances, induction of the pET:Bb-Eno1 clone by IPTG resulted in the production of two bands, the first having the expected mass of 47 kDa and a second smaller band with a mass ≈45 kDa (Fig 1, lane 2). Similarly, the Bb-F2 clone also appeared to produce two protein bands of ≈28 kDa (Figure 1, lane 4). Further experimentation revealed that these bands were due to cleavage during heat denatuation (Fig 3).

### IgE immunoblot analysis of recombinant proteins

**Figure 4:**
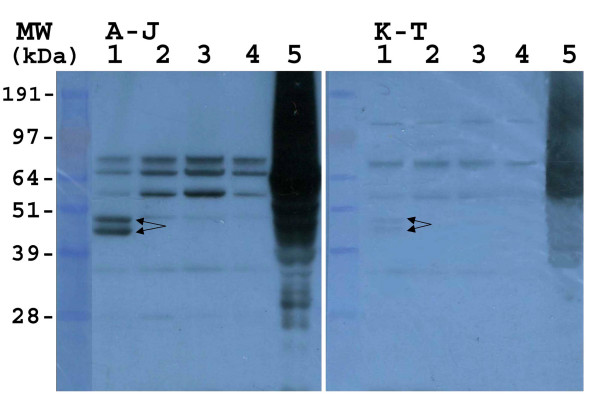
Immunoblot analysis of recombinant *B. bassiana *proteins. Immunoblots were probed with sera pooled from (10 each) patients displaying mold allergies as indicated on the panels (A-J, and K-T). The final concentration of individual sera in each pool was 1:35. An HRP conjugated goat anti-human IgE antibody was used as the secondary antibody. Lanes contain recombinant *E. coli *expressed proteins as follows, lane 1) Bb-Eno1, 2) Bb-f2,. 3) Bb-Ald, 4) Bb-Hex. Lane 5) 40 μg crude *B. bassiana *extract.

Immunoblots were used in order to determine whether human IgEs could bind the recombinant *B. bassiana *proteins. Crude *E. coli *extracts containing the expressed proteins were resolved by SDS-PAGE and transferred to PVDF membranes as described in the Methods. Initial experiments were performed using blots containing the four expressed proteins as well as a crude *B. bassiana *extract (positive control), that were probed with one of two sera pools containing serum from ten patients each, pools A-J and K-T (Fig. 4). Each blot was treated with 0.2 ml of each serum (1:35 dilution, final concentration). The blot probed with pool A-J revealed strong IgE binding of the two protein bands corresponding to BbEno1, as well as several reactive (background) *E. coli *bands. The *B. bassiana *crude extract reacted with a variety of IgEs present in the sera as has been previously reported. From the sera tested, faint IgE binding to Bb-Ald was noted, with no visible IgE binding observed for Bb-f2 and Bb-Hex. Control blots using *E. coli *crude extracts derived from cells harboring the vector with no insert resulted in essentially the same background bands as seen with extracts containing the expressed proteins.

**Figure 5:**
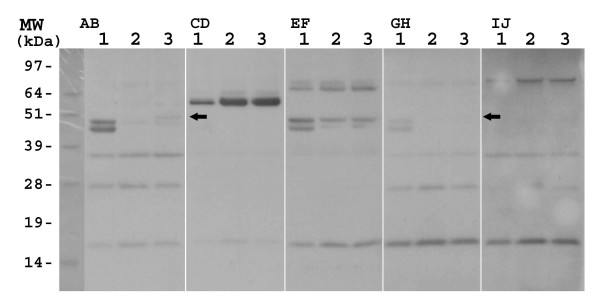
Immunoblot analysis of recombinant *B. bassiana *proteins. Immunoblots were probed with sera pools (2 each) as indicated on the panels (AB, CD, EF, GH, and IJ), with a 1:5 final concentration of individual sera in each pool. Blots were probed with an HRP conjugated goat anti-human IgE antibody as the secondary antibody. Lanes contain recombinant *E. coli *expressed proteins as follows, lane 1) Bb-Eno1, 2) Bb-f2,. 3) Bb-Ald.

**Figure 6:**
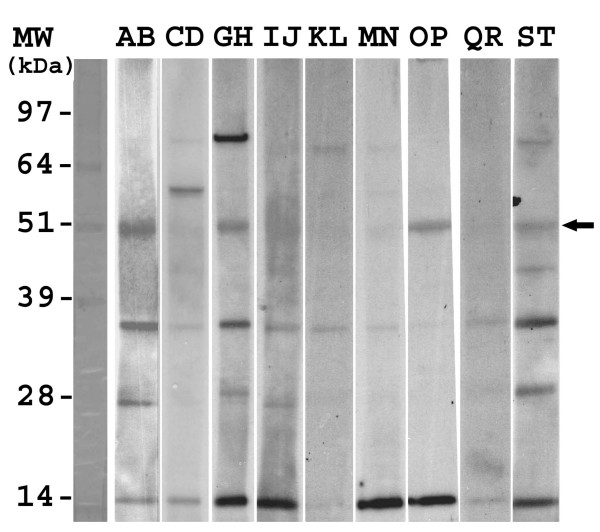
Immunoblot analysis of recombinant Bb-Ald. PVDF membrane strips containing crude extracts of *E. coli *expressed Bb-Ald were probed with 1 mL of each designated sera pool, with each pool containing two sera (final dilution 1:5 each sera). Arrow indicates the position of Bb-Ald.

**Figure 7:**
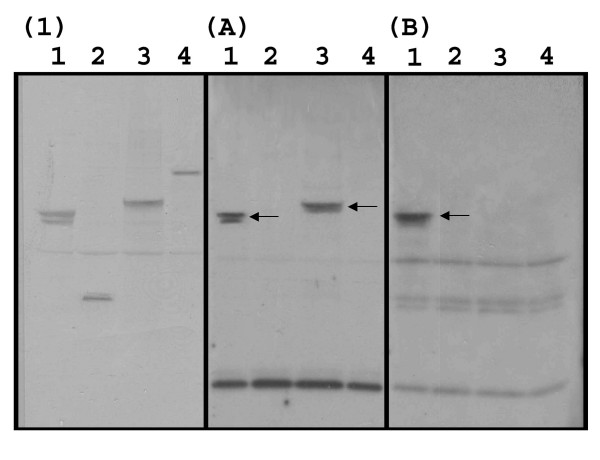
Ponceau S staining and immunoblot analysis of recombinant *B. bassiana*. Immunoblots were probed with individual sera, A) and B) as indicated on the panels using a 1:5 final concentration of sera. Blots were probed with an HRP conjugated goat anti-human IgE antibody as the secondary antibody. Lanes contain recombinant *E. coli *expressed proteins as follows, lane 1) Bb-Eno1, 2) Bb-f2,. 3) Bb-Ald, and 4) Bb-Hex. Panel 1) represents Ponceau S staining of the PVDF membrane after transfer.

In order to confirm the binding of IgEs to Bb-Ald, additional experiments using smaller sera pools and higher final concentrations of individual sera were performed. Five sera pools, each containing 1:5 dilutions of two sera, and designated as AB, CD, EF, GH, IJ, KL, MN, OP, QR, and ST were created. In one set of experiments pools AB, CD, EF, GH, and IJ were then used to probe membranes containing Bb-Eno1, Bb-f2, and Bb-Ald, (Fig. 5, Bb-Hex was omitted due to the lack of reactivity in preliminary experiments). These results confirmed IgE binding to Bb-Eno1 (strong signal from pools AB and EF, with weaker signal from pool GH) and to Bb-Ald (pools AB and GH). Not too surprisingly, IgE binding of "background bands", *i.e*. antigens derived from the *E. coli *extracts were highly variable between pools. Using these sera pools, no IgE binding was observed to Bb-f2. In a second series of experiments the pools were used to probe membrane strips containing only Bb-Ald extracts. IgE binding of Bb-Ald was noted using pools AB, GH, OP, and ST (Fig. 6). Since pool AB resulted in strong signals to both Bb-Eno1 and Bb-Ald, further experiments were performed using the individual sera (either A or B) to probe membranes containing all four *B. bassiana *recombinant proteins (Fig 7). These results revealed that serum A contained IgEs that bound to Bb-Eno1 and Bb-Ald, whereas serum B contained IgEs reactive only to Bb-Eno1.

### Phylogenetic analyses

**Figure 8:**
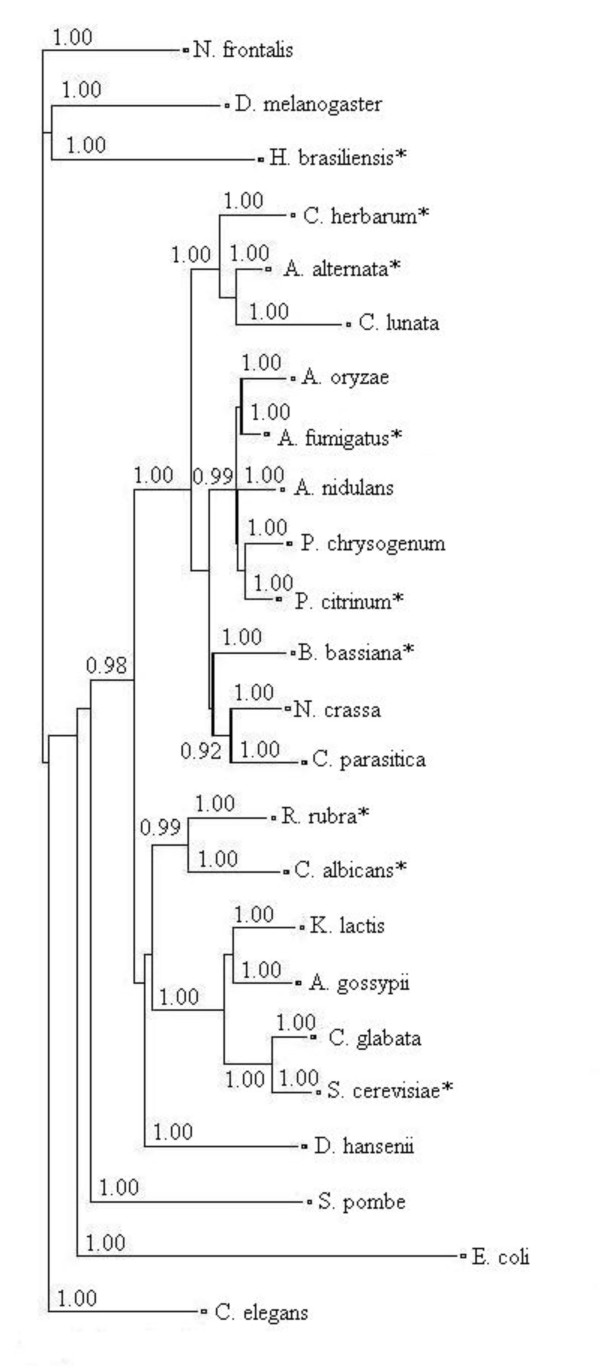
Full length amino acid sequences of 24 enolases deposited in the NCBI Genbank database were used to construct an enolase phylogram. Normalized posterior probabilities values greater than or equal to 0.9 are presented at their respective nodes. Known allergenic enolases are denoted by an asterisk.

Bb-Eno1 displayed high sequence similarity to fungal enolases several of which are known allergens. A phylogram was constructed using the amino acid sequences of 21 fungal enolases as well as those of *Drosophila melanogaster*, *E. coli*, and the rubber plant, *Hevea brasiliensis*, a known potent allergen (Fig. 8). Of the enolases examined, nine have been identified as allergens (designated with an asterisk in the figure). These proteins do not appear to cluster in any discernable pattern and are equally distributed throughout the phylogram. Similarly, an analysis of the available fungal aldehyde dehydrogenases failed to reveal any discernable pattern or clustering of the known allergens.

## Discussion

Allergy is a hypersensitive response of the immune system and fungi are important triggers of respiratory and other forms of allergies [5,33-35]. As alternatives to chemical pesticides, entomopathogenic fungi such as *Metarhizium anisopliae *and *Beauveria bassiana *hold promise as biological control agents, and both organisms have been EPA approved for commercial control of a variety of arthropod pests [8-11]. The process of fungal infection of insect targets involves the use of infectious propagules, typically (conidia) spores, which attach and germinate across host surfaces. Growing fungal cells then begin to penetrate the cuticle and proliferate within the insect body, ultimately resulting in the death of the host [6,36,37]. Use of these biological pesticides, however, is likely to lead to the dispersal of inhalable fungal particles. Several studies have demonstrated the potential of these fungi in eliciting allergic reactions. [12,14,38]. Some occupational allergy to *M. anisopliae *has been noted and immune and pulmonary responses characteristic of allergy were observed in Balb/c mice challenged with *M. anisopliae *extracts [39,40]. Furthermore, allergen-triggered airway hyperresponsiveness and lung pathology occurred in mice sensitized with this fungus [41]. The allergenic potential of *B. bassiana *has been confirmed by intradermal skin testing, and numerous IgE reactive proteins, some of which are cross-reactive among allergens from other fungi have been noted in this organism [14]. To date, however, there have been no reports detailing the molecular identification of *B. bassiana *(or *M. anisopliae*) IgE-reactive antigens.

The present study describes the cloning and expression of four putative *B. bassiana *allergens and demonstrated IgE-reactivity for two of the recombinant proteins using sera derived from patients displaying mold allergies. Bb-Eno1 has a calculated monomer molecular mass of 47.4 kDa and displays similarity to enolases that form an extensively studied group of allergens. IgE cross reactivity between the enolases of *C. herbarum*, *A. alternata*, *C. albicans*, and *A. fumigatus *has been well characterized and it is likely that the *B. bassiana *protein would also be recognized by the same IgEs. Modeling of the *C herbarum *enolase using the solved crystal structure of the *S. cerevisiae *enolase was used to construct 10 recombinant peptides spanning the length of the *C. herbarum *enolase [42]. Six of these peptides, distributed throughout the entire length of the protein showed IgE-binding activity. One of the peptides encompassed a region that overlapped with the other 5 IgE reactive peptides and formed, based upon the modeling, an extended structure that twice spanned the body of the globular protein and reached the surface three times. This sequence was therefore deemed contain at least one immunodominant IgE epitope, and sequence analyses revealed a highly similar stretch of amino acids in the deduced *B. bassiana *enolase sequence. Approximately 20% of the sera tested (4–6/20) displayed positive IgE reactivity to the recombinant Bb-Eno1, indicating that this protein is likely to be a significant allergen in *B. bassiana*.

Bb-Ald was similar to the *A. alternata *Alt a 10 and *C. herbarum *Cla h 3 proteins, both of which have been characterized as aldehyde dehydrogenases [18]. In a survey of allergens recognized by patients with mold allergies, 2% displayed IgE reactivity to Alt a 10, whereas 36% displayed reactivity to Cla h 3 [18]. Based upon these results, Cla h 3 was classified as an important allergen and Alt a 10 as a minor allergen. Only one (out of twenty) of our sera displayed strong reactivity to Bb-Ald, indicating that this protein is indeed an allergen, however due to our small sample size, it is not possible to draw any definitive conclusions regarding the importance of this protein as a *B. bassiana *allergen.

Bb-f2 showed sequence homologies to the *A. fumigatus *Asp f2 major allergen and the fibrinogen binding protein from *C. albicans *[43]. In *A. fumigatus*, Asp f2 appears to be expressed as a 55-kDa mycelial glycoprotein as well as a 37-kDa culture filtrate presumably deglycosylated protein (the calculated molecular mass of Asp f2 is 29 kDa, the reason for the discrepancy is unclear but may be attributed to specific C-terminal amino acid residues), both of which are IgE reactive [31]. Asp f2 also appears to interact with extracellular matrix proteins such as laminin, and exhibited IgE binding from sera derived from patients with allergic bronchopulmonary aspergillosis (ABPA) and cyctic fibrosis-ABPA patients, but not from sera isolated from *A. fumigatus*-sensitized allergic asthma (and normal control subjects) [31]. Thus, the observed lack of IgE reactivity to Bb-f2 may be attributed to the lack of ABPA patients in our sera samples.

The original cDNA fragment corresponding to Bb-Hex displayed the highest similarity to the N-acetylhexosaminidase of *P. chrysogenum *(Pen ch 20), that has been identified as an allergen [32]. Subsequent, full length cDNA cloning and characterization resulted in higher similarity to other fungal hexosaminidases that have not been characterized as allergens (see E-values in Table 2). None of our sera samples displayed IgE reactivity to recombinant Bb-Hex, however, due to our low sample size the possibility cannot be excluded that this protein represents a *B. bassiana *allergen.

Our results confirm the potential allergenicity of *B. bassiana *by the molecular and immunological characterization of specific allergens from this organism and suggest that some precautions should be taken into account in biological control applications using entomopathogenic fungi. It is, however, important not to overstate the potential risks (versus benefits) and the overall safety with respect to allergenicity of these fungi may be similar to that of baker's yeast from which allergens, including enolase, have also been isolated [44,45]. While it is not possible to determine the fraction of the total allergen production the four isolated proteins represents, immunoblot comparisons between the isolated proteins and the reactivity of crude *B. bassiana *extracts indicated the presence of numerous additional allergens that have yet to be characterized, some of which may represent more highly antigenic epitopes. The isolation of putative *B. bassiana *allergens described in this report relied upon identifying molecules by the resemblance of their DNA sequences to previously identified allergens and future experiments using alternate approaches (*e.g*. phage display [46]) may be needed to identify additional allergens. Finally, although much work has been performed in regards to isolating and characterizing fungal allergens, the roles of these proteins in fungal processes such as development and pathogenesis remains obscure. As a genetically amenable organism and a pathogen of arthropods, *B. bassiana *represents a novel system to examine the relationship between allergenicity and (insect) pathogenesis. Targeted gene-knockouts, for instance, can be used to probe affects upon virulence and interactions between specific allergen and arthropod innate immune systems.

## Conclusion

Cloning, sequencing, and heterologous expression of four putative *B. bassiana *allergens was performed. Recombinant proteins corresponding to Bb-Eno1 and Bb-Ald, but not Bb-f2 and Bb-Hex, displayed IgEs reactivity against sera from patients with mold allergies. Due to the low sera sample numbers used, it cannot be excluded that Bb-f2 and Bb-Hex are allergens, and further testing is warranted. Bb-Eno1 was similar to enolases that represent a well characterized group of major allergens. Bb-Ald was similar to aldehyde dehydrogenases that are considered major allergens in some fungal species, but minor allergens in others. The molecular identification of *B. bassiana *allergens can lead diagnostic methods for determining sensitization to this organism and provides a rational basis for allergen attenuation in order to yield safer biological control products. The *B. bassiana*-arthropod interaction may represent a novel model system to examine the relationships between allergenicity and pathogenicity.

## Abbreviations

ABPA, allergic bronchopulmonary aspergillosis, BLAST, basic local alignment search tool, EPA, Environmental Protection Agency, E-value, expect score, EDTA, ethylenediaminetetraacetic acid, EST, expressed sequence tags, HRP, horseradish peroxidase, IgE, immunoglobulin E, IPTG, isopropyl-b-D-thiogalactoside, LB, Luria-Bertani broth, NCBI, National Center for Biotechnology Information, PAGE, polyacrylamide gel electrophoresis, PCR, polymerase chain reaction, PMSF, phenylmethyl sulfonyl fluoride, PVDF, polyvinylidene fluoride, RACE, rapid amplification of cDNA ends, SDS, sodium duodecyl sulfate, SSH, suppressive subtractive hybridization, TBS, Tris buffered saline.

## Competing interests

The author(s) declare that they have no competing interests.

## Authors' contributions

GSW carried out the molecular, immunological, and other *in vitro *experiments, and participated in the design of the study. SWH participated in the design of the study and provided technical support for the project. NOK conceived of the study, participated in its design and coordination, and drafted the manuscript.
